# *Aleuritopteris hainanensis* (Pteridaceae), a New Species From Hainan, China

**DOI:** 10.1002/ece3.72770

**Published:** 2026-02-20

**Authors:** Bin Zhang, Ting Wang, Gui‐Liang Zhang, Guo‐Hua Zhao, Zi‐Yue Liu, Fa‐Guo Wang, Yue‐Hong Yan, Hong‐Feng Chen

**Affiliations:** ^1^ State Key Laboratory of Plant Diversity and Specialty Crops, South China Botanical Garden Chinese Academy of Sciences Guangzhou Guangdong China; ^2^ University of Chinese Academy of Sciences Beijing China; ^3^ Hekou Branch of Management and Protection Bureau of Daweishan National Nature Reserve Hekou Yunnan China; ^4^ Shenzhen Key Laboratory of Southern Subtropical Plant Diversity Fairy Lake Botanical Garden, Shenzhen & Chinese Academy of Sciences Shenzhen Guangdong China; ^5^ Eastern China Conservation Centre for Wild Endangered Plant Resources, Shanghai Chenshan Botanical Garden Shanghai China

**Keywords:** Cheianthoideae, ferns, phylogeny, taxonomy

## Abstract

*Aleuritopteris hainanensis*, a new fern of Pteridaceae from Hainan Province, China, is described and illustrated. Morphologically, *A. hainanensis* closely resembles *A. squamosa* , with both species bearing densely scaly fronds. However, the fertile fronds of *A. squamosa* (10.7–25.9 cm) are significantly longer than the sterile fronds (4.1–10.7 cm), whereas in *A. hainanensis* , the sterile (9.4–13 cm) and fertile fronds (10.1–14.1 cm) are nearly equal in length. In addition, the stipe and abaxial lamina scales of *A. hainanensis* are broadly lanceolate with nearly entire margins, while those of *A. squamosa* are lanceolate with margins erose‐serrulate. Plastomes analysis futher reveals that the genetic distance between *A. hainanensis* and *A. squamosa* is significantly greater than the typical intraspecific variation, supporting the recognition of *A. hainanensis* as a distinct species. According to IUCN criteria, we propose that *A. hainanensis* should be categorized as a Vulnerable (VU) species.

## Graphical abstract

A new fern species, *Aleuritopteris hainanensis*, is described from Hainan Province, China, closely resembling *A. squamosa* but differing in fertile/sterile frond length and scale morphology. Plastome analysis confirms its distinctness, showing significant genetic divergence from *A. squamosa* . The species is proposed to be classified as Vulnerable (VU) under IUCN criteria.

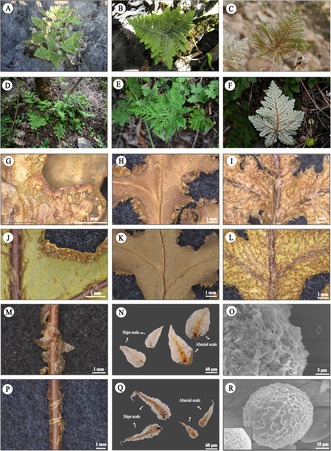

## 1 Introduction

*Aleuritopteris* Fée (1852, 153) is a genus of ferns in the subfamily Cheilanthoideae of the Pteridaceae, originally established based on *Aleuritopteris farinosa* (Forssk.) Fée (≡ *Pteris farinosa* Forssk., 1775, 187). It is widely distributed across tropical and subtropical regions of both the Old and New Worlds (Zhang and Yatskievych 2013). In recent classifications, several previously segregated genera, including *Oeosporangium* Vis. (1867, 663), *Mildella* Trevis. (1877, 810), *Leptolepidium* K. H. Shing & S. K. Wu (1979, 115), *Negripteris* Pic. Serm. (1946, 130), and *Sinopteris* C. Christensen & Ching (1933, 359), have been incorporated into *Aleuritopteris*, significantly expanding its circumscription (Schuettpelz et al. 2025). Currently, *Aleuritopteris* comprises approximately 100 species worldwide (Schuettpelz et al. 2025), with 44 species recorded in China.

Despite this taxonomic progress, species delimitation within *Aleuritopteris* remains challenging owing to extensive morphological convergence, phenotypic plasticity, and overlapping character states. For instance, many herbarium specimens of *Aleuritopteris* collected from Hainan, China (e.g., PE 00539557, PE 00539558; IBSC 705509, IBSC 705511; SZG 00031312; HUST 00000049, HUST 0006765, HUST 00006766) have frequently been misidentified as *A. squamosa* (C. Hope & C. H. Wright) Ching (1903, 518) because of their superficial morphological similarities. However, through meticulous examination of all type specimens of *A. squamosa* and relevant herbarium materials, combined with detailed morphological comparisons and phylogenetic analyses, we identified that the *Aleuritopteris* specimens from Hainan represent a species distinct from *A. squamosa* . Accordingly, we described the Hainan population here as a new species, *Aleuritopteris hainanensis* sp. nov.

## 2 Materials and Methods

### 2.1 Morphological Analysis

Specimens from the herbaria IBSC, PE, SZG, HUST and K, were examined, observed, and measured for morphological comparison. The voucher specimens of *A. hainanensis* are deposited at South China Botanical Garden Herbarium (IBSC 0672304 and 0672305). Stipe and abaxial scales were observed using an OLYMPUS‐SZ61 stereoscopic microscope and an OLYMPUS‐BX43 biological microscope. Morphological data of *A. hainanensis* (12 fertile and 8 sterile fronds) and *A. squamosa* (11 fertile and 7 sterile fronds) were measured with MATO (Liu et al. 2023). The ornamentation of spores was scanned by JSM‐IT210LV Scanning Electron Microscope.

### 2.2 Taxon Sampling, DNA Extraction, and Sequencing

In this study, three new *Aleuritopteris* plastomes were sequenced and completely assembled. All leaf samples are stored in silica gel. Total DNA was extracted from the leaves using a modified CTAB method and sequenced using short reads produced by the DNBSEQ platform (2 × 150 bp) by BGI (Shenzhen, China). Comprehensive sample information is shown in Table 1.

**TABLE 1:** List of vouchers used in this study.

GenBank no.	Species	Collection no.	Herbarium	Locality
PX841687	*Aleuritopteris hainanensis*	0004645	SZG	China, Hainan, Changjiang
PX841685	*Aleuritopteris squamosa*	WT202417	IBSC	China, Yunnan, Gejiu
PX841686	*Aleuritopteris squamosa*	WT202419	IBSC	China, Yunnan, Gejiu

### 2.3 Plastome Assembly, Annotation, and Comparison

The raw data of each sample were quality‐filtered using Trimmomatic v.0.36 (Bolger et al. 2014) with default parameters. The resulting high‐quality, paired‐end reads were assembled into contigs using the GetOrganelle pipeline (https://github.com/Kinggerm/GetOrganelle) with the parameters set as R (Maximum extension rounds) = 15 and *k* (kmers) = 21, 45, 65, 85, 105. The assembled plastomes were visually inspected and edited using Bandage (Wick et al. 2015), then a complete circular plastome was generated for each sample. The plastomes were annotated with reference to *Cheilanthes micropteris* (MH173078) in Geneious v.11.1.5 (Kearse et al. 2012). The sequence comparison among the two species was detected using mVISTA (Frazer et al. 2004) software with the default settings, and *A. squamosa* was chosen as the reference.

In addition, to verify the presence of enlarged noncoding regions (Kim 2024), gap distributions across the MAFFT‐aligned plastome sequences of *A. hainanensis* and *A. squamosa* were quantified. Gaps were first converted into binary vectors (gap = 1, nucleotide = 0) using the “Biostrings” package, and then a 1000‐bp sliding window was applied using the “zoo” package to calculate the proportion of gaps within each window along the alignment (Arimon‐Pagès et al. 2019; Zeileis and Grothendieck 2007) in R 4.3.1 (R Core Team 2023).

### 2.4 Phylogenetic Analyses

In this study, we analyzed the plastid phylogenetic relationships of *Aleuritopteris* species by combining newly generated data with publicly available complete plastome sequences from the National Center for Biotechnology Information (NCBI; https://www.ncbi.nlm.nih.gov/). To include a broader sampling of species within the genus, we also constructed a separate dataset based on the *rbcL* gene for phylogenetic inference. Detailed information on the samples used is provided in Tables S1 and S2. Sequence alignment was performed using MAFFT v.7.475 (Katoh and Standley 2013) and poorly aligned regions were trimmed with trimAl (Capella‐Gutiérrez et al. 2009).

We used maximum likelihood (ML) and Bayesian inference (BI) methods for phylogenetic construction. The best‐fit model of evolution of ML and BI methods was selected by ModelTest‐NG (Darriba et al. 2020) under the Bayesian Information Criterion (BIC) with “‐T raxml” and “‐T mrbayes” parameters, respectively. ML analyses were conducted with RAxML v.8.2.10 (Stamatakis 2014) and node support was assessed using rapid bootstrap (RBS) analysis with 1000 pseudo‐replicates. BI analyses were constructed with MrBayes v.3.2 (Ronquist et al. 2012), using 1 million generations and sampling trees every 100 generations. Two runs each with three heated and one cold chain were performed in parallel. Each chain started with a random tree and the first 25% of sampled generations were discarded as burn‐in to construct a majority‐rule consensus tree and estimate the posterior probabilities (PP). The convergence of runs was assumed when the average standard deviation of split frequencies dropped below 0.01 according to the MrBayes manual.

### 2.5 Genetic Distance Estimation

The intraspecific and interspecific genetic distances for both complete plastomes and *rbcL* sequences of *Aleuritopteris* were calculated using the two‐parameter (K2P) model in MEGA 11.0 (Tamura et al. 2021). Subsequently, the “ggplot2” (Wickham 2011) and “ggpubr” (https://rpkgs.datanovia.com/ggpubr/) packages in R were employed to analyze and visualize potential significant differences between the intraspecific and interspecific genetic distances using the Wilcoxon test.

## 3 Results

### 3.1 Comparison of *A. hainanensis* and *A. squamosa* Plastomes

The plastome of *A. hainanensis* is 150,647 bp in length, compared to 155,428 and 155,437 bp in two *A. squamosa* specimens. All plastomes exhibit the typical conserved quadripartite structure, comprising a large single‐copy (LSC) region, a small single‐copy (SSC) region, and a pair of inverted repeats (IRs). The GC content ranges from 40.9% to 41.3%.

**FIGURE 1:**
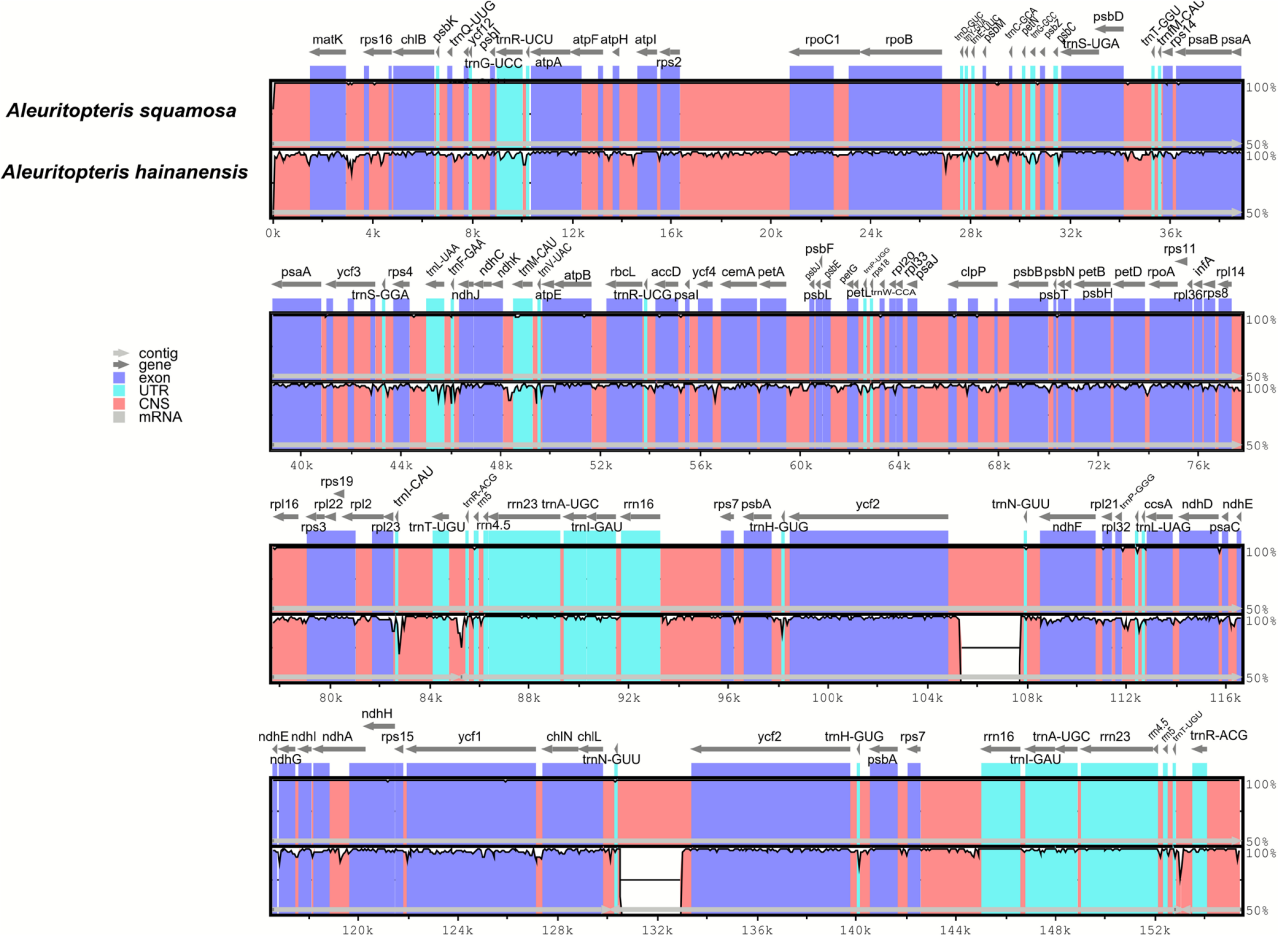
Comparison in plastome sequence alignment of *Aleuritopteris hainanensis* and *Aleuritopteris squamosa* . The *y*‐axis represents the percentage identity (50%–100%), and the *x*‐axis shows the position of each gene. Gray arrows above the alignment indicate the transcriptional directions of genes. Genome regions are color‐coded as exon, untranslated region (UTR), and conserved noncoding sequences (CNS).

Through whole‐sequence alignment and visual analysis of *A. hainanensis* and *A. squamosa* plastomes using mVISTA online tool, we found that the two plastomes share highly similar gene contents (Figure 1). Despite this overall similarity, several regions display notable sequence divergence. Most sequence variations are located in the intergenic regions, such as *trnN‐GUU‐ycf2*, *rpoB‐trnD‐GUC*, *ycf4‐CemA*, *trnT‐UGU‐trnR‐ACG*. In addition, compared with *A. squamosa*, *A. hainanensis* shows conspicuously enlarged gap regions in trnN‐GUU–ycf2 (Figure S1). These enlarged gaps correspond to enlarged noncoding regions in fern organelles (ENRFOs) within foreign DNA insertions present in the plastome of *A. squamosa*.

### 3.2 Phylogenetic Relationships and Genetic Distance Within *Aleuritopteris*

**FIGURE 2:**
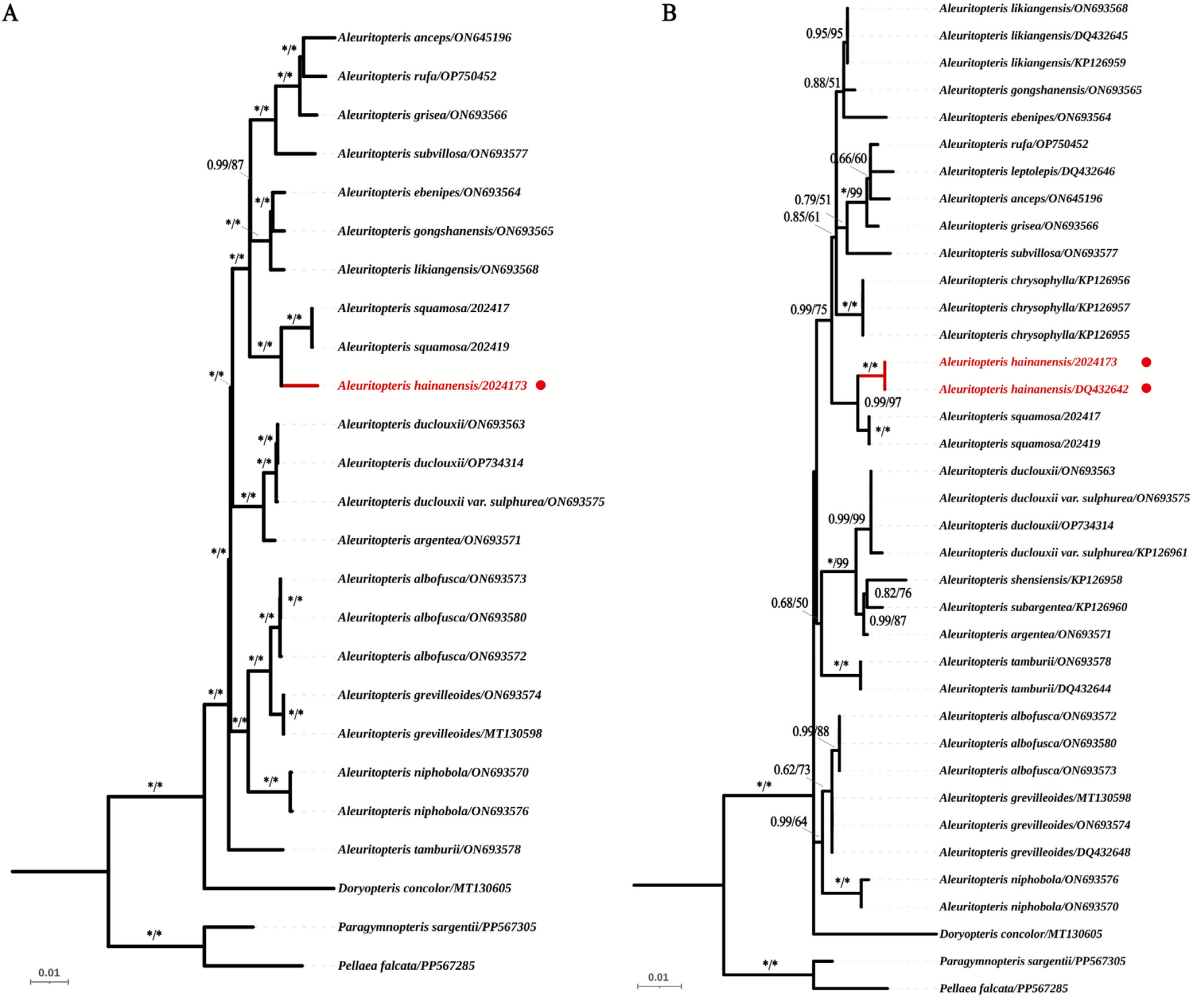
Maximum likelihood and Bayesian inference tree of *Aleuritopteris* species, based on (A) complete plastid genomes and (B) rbcL sequences. Maximum likelihood tree and Bayesian tree only show nodes with bootstrap values > 50%. * represent 100%. Nodes marked in red represent newly discovered species.

**FIGURE 3:**
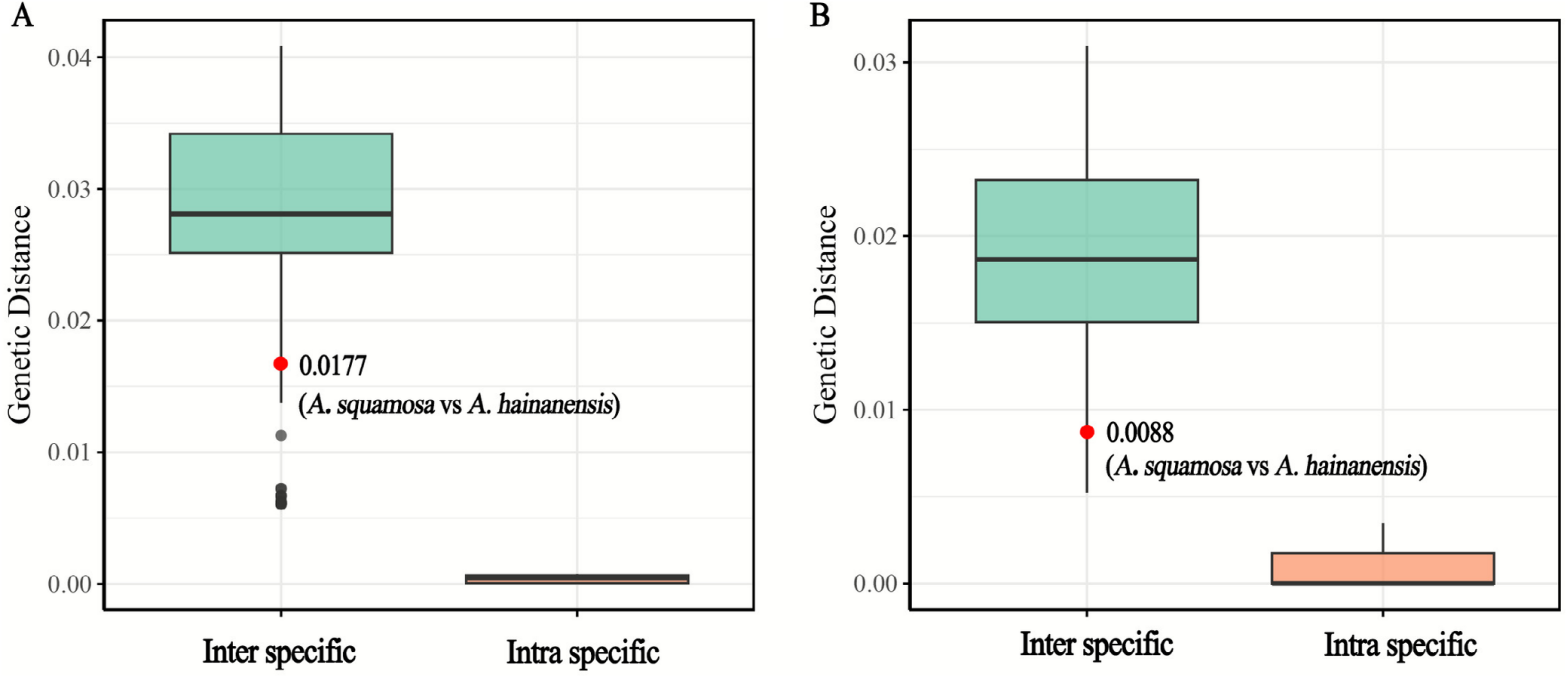
Intraspecific and interspecific genetic distance of *Aleuritopteris*. (A) Plastome and (B) *rbcL*. Detailed information can be found in Tables S3 and S4.

Bayesian inference (BI) and maximum likelihood (ML) phylogenetic trees were constructed based on both complete plastomes and *rbcL* genes (Figure 2). The resulting phylogenies revealed identical topologies, with *A. hainanensis* and *A. squamosa* forming a sister relationship, supported by high bootstrap (RBS = 100%) and posterior probability values (PP = 100%). In terms of genetic distance within *Aleuritopteris*, the intraspecific plastome divergence ranges from 0.000037 to 0.000762, whereas the interspecific plastome divergence ranges from 0.017685 to 0.102426. For the *rbcL* gene, intraspecific divergence ranges from 0.000000 to 0.001735, and interspecific divergence ranges from 0.008769 to 0.072160. The genetic distance between *A. hainanensis* and *A. squamosa* is 0.017685 for the plastome and 0.008769 for *rbcL*, which falls within the range of interspecific divergence (Tables S3 and S4; Figure 3). Collectively, these results indicate that although *A. hainanensis* is closely related to *A. squamosa* , the genetic distance between them is comparable to that observed among distinct species, thereby supporting the recognition of *A. hainanensis* as a distinct species rather than conspecific with *A. squamosa* .

### 3.3 Taxonomic Treatment

**FIGURE 4:**
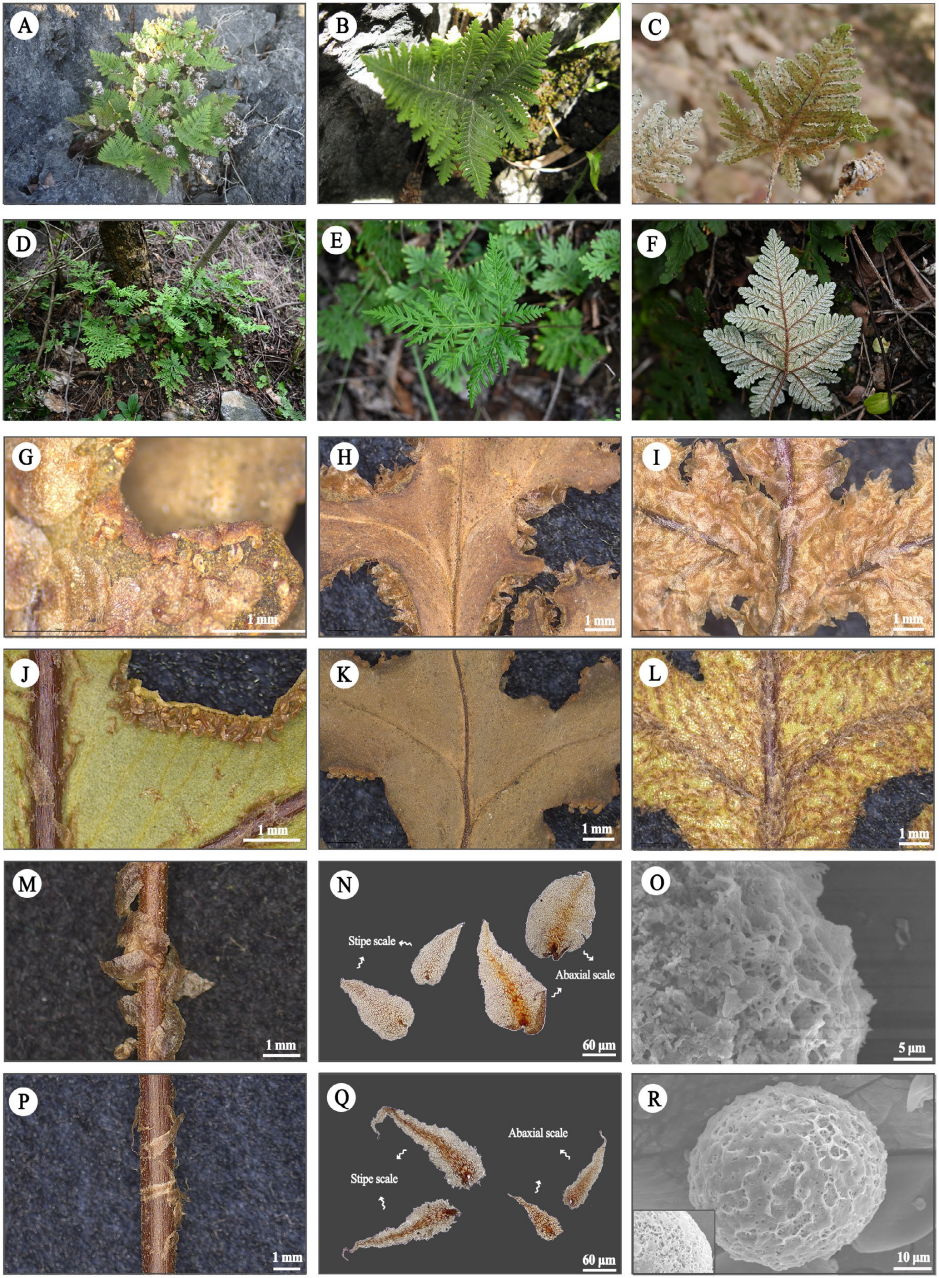
Morphological comparison of *Aleuritopteris hainanensis* and *Aleuritopteris squamosa*. *Aleuritopteris hainanensis*: (A–C) plant, (G) false indusia, (H) lamina, (I) abaxial scales, (M) stipe scales, (N) scales, (O) spores. *Aleuritopteris squamosa*: (D–F) plant, (J) false indusia, (K) lamina, (L) abaxial scales, (P) stipe scales, (Q) scales, (R) spores.

**FIGURE 5:**
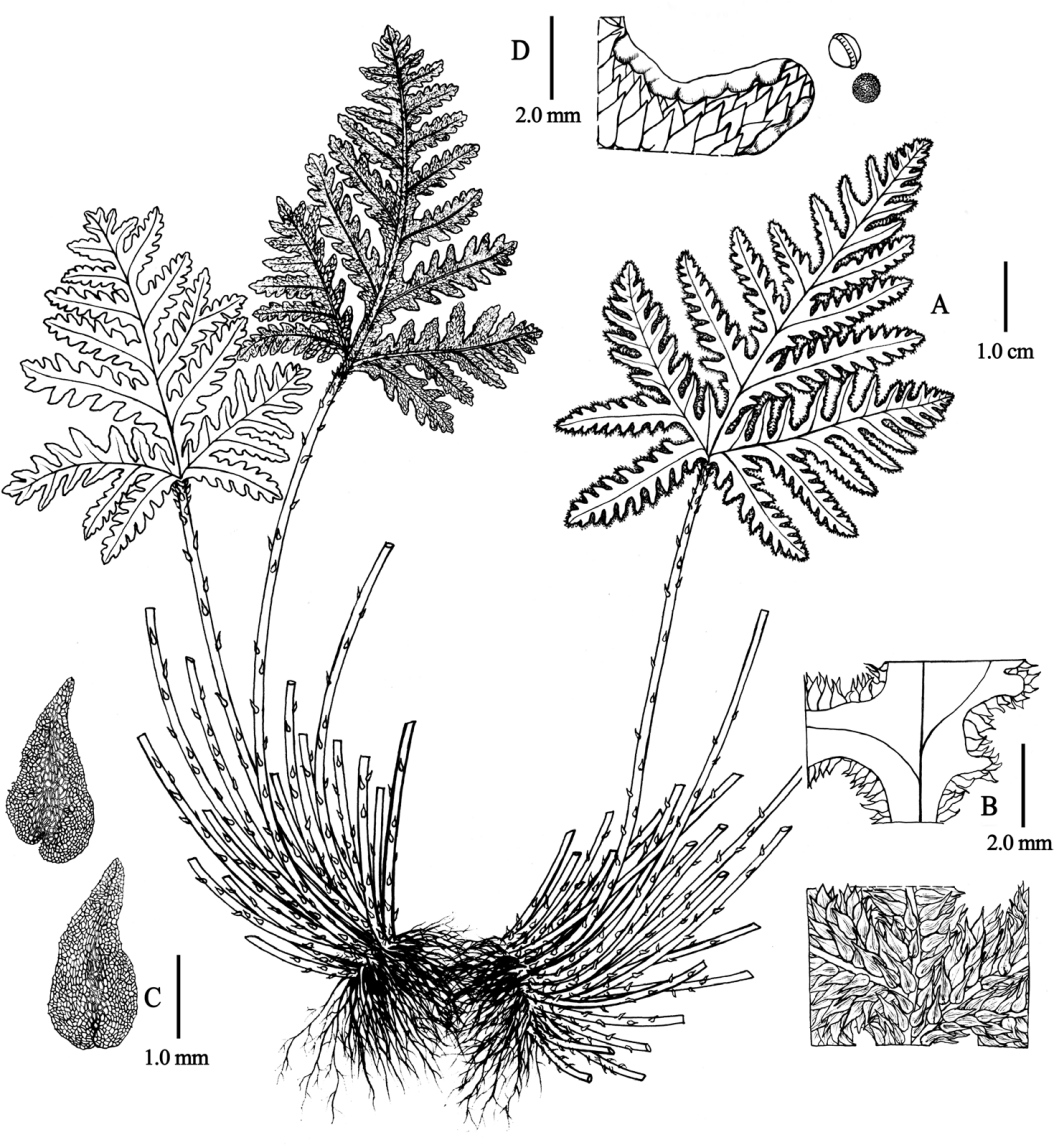
*Aleuritopteris hainanensis*. (A) Plant; (B) lamina and abaxial scales; (C) scales; (D) false indusia and spores. Illustrated by Yun‐Xiao Liu.

**Aleuritopteris hainanensis Bin Zhang, Ting Wang tris & H. F. Chen, sp. nov**. Figures 4 and 5.

**Type**. China. Hainan: Changjiang Li Autonomous County, Bawang Ling, ca. 300–600 m elev., 25 March 1989, Fu‐wu Xing et Ze‐xian Li (holotype: IBSC [0672305!], isotype: IBSC [0672304!]).

**FIGURE 6:**
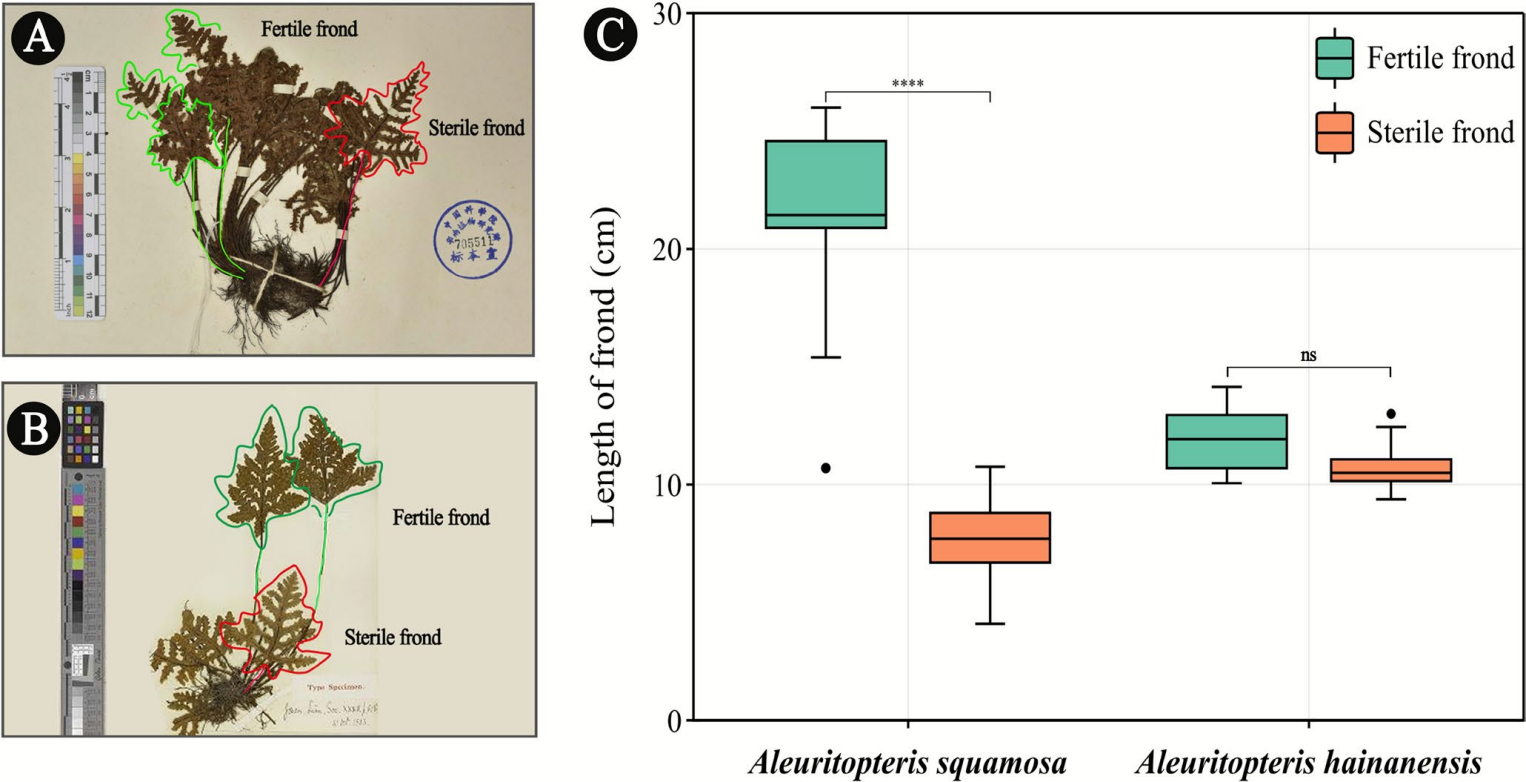
Comparison of fertile and sterile frond lengths between *Aleuritopteris hainanensis* and *Aleuritopteris squamosa* . (A, B) Representative type specimens of *A. hainanensis* (IBSC 0672305) and *A. squamosa* (K 001057912). Green and red lines represent fertile fronds and sterile fronds, respectively; (C) Boxplot comparing the lengths of fertile and sterile fronds between the two species. Asterisks (****) denote statistically significant differences (*p* < 0.001), “ns” indicates no significant difference (*p* ≥ 0.05).

**Diagnosis**. *Aleuritopteris hainanensis* is morphologically quite similar to *A. squamosa* , with both species bearing densely scaly fronds. However, our observations reveal that in *A. hainanensis* , the sterile and fertile fronds are nearly equal in length (Table 2; Figure 6), whereas in *A. squamosa* , the fertile fronds are significantly longer than the sterile ones (*p* < 0.05), a characteristic that has not been explicitly recorded in the *Flora of China* or *Flora Yunnanica* (Chu et al. 2006; Zhang and Yatskievych 2013). In addition, the stipe and abaxial lamina scales of *A. hainanensis* are broadly lanceolate with nearly entire margins (occasionally bearing minute teeth; Figure 4N), while those of *A. squamosa* are lanceolate with coarsely toothed margins (Figure 4Q). The genetic distance analysis also supports the recognition of *A. hainanensis* and *A. squamosa* as distinct species (Figure 3).

**TABLE 2:** Morphological comparison of *Aleuritopteris hainanensis* and *Aleuritopteris squamosa*.

Characters	*Aleuritopteris hainanensis*	*Aleuritopteris squamosa*
Fertile frond	10–14 cm	10–26 cm
Sterile frond	9–13 cm	4–11 cm
Stipe scales	Brown, broadly lanceolate, nearly entire	Brown, lanceolate, erose‐serrulate
Lamina	Pentagonal 3–5 cm × 3–5 cm; abaxial surface with broadly lanceolate scales	Pentagonal 5–10 cm × 5–10 cm; abaxial surface with lanceolate scales
Pinnae	5–9 pairs	5–7 pairs
False indusia	Continuous, membranous, margins entire	Continuous, membranous, margins entire
Spores	Reticulate	Reticulate

**Description**. *Plants* terrestrial. *Rhizomes* suberect to ascending, short. *Fronds* clustered. Fertile frond 10–14 cm, sterile frond 9–13 cm. *Stipe and rachis* dark brown, shiny, terete, and densely covered with broadly lanceolate, nearly entire‐margined, bicolorous scales with brown central stripe and lighter margins. *Lamina* brownish green, pentagonal, nearly as long as broad, 3–5 cm × 3–5 cm, 3‐pinnatipartite, thickly papery when dry, abaxially covered with snow‐white farina and densely with scales similar to those on the stipe; adaxially glabrous; apex shortly acuminate. *Pinnae* 5–9 pairs, connected by a narrow wing; basal pair largest, subtriangular, 2–3 cm, obliquely spreading upward, 2‐pinnatipartite; pinnules 4–7 pairs, second and upper pairs of pinnae gradually shortened distally, lanceolate or oblong‐lanceolate, pinnatipartite, base decurrent and connected to rachis by broad wings, apex shortly acute. *False indusia* continuous, narrow, membranous, margins entire. *Spores* exhibit reticulate ornamentation, with 52–62 μm in diameter.

**Additional specimens examined**: CHINA. Hainan: Changjiang Li Autonomous County, Shilu Town, ca. 350 m elev., 2 April 2002, Zhen‐Chuan Chen (SZG 00031312). Changjiang Li Autonomous County, Qicha Village, ca. 380 m elev., 2 April 2002, Xian‐Chun Zhang (PE 00539558). Changjiang Li Autonomous County, Qicha Village, ca. 375 m elev., 2 April 2002, Shi‐Yong Dong (PE 00539557). Changjiang Li Autonomous County, Wangxia Village, ca. 600 m elev., April 2003, Yue‐Hong Yan (HUST 00000049). Dongfang City, Tianan Village, May 2003, Xin‐Sheng Qin (HUST 00006765). Dongfang City, Jiangbian Village, ca. 300 m elev., May 2003, Xin‐Sheng Qin (HUST 00006766).

**Geographical distribution**. Currently, *A. hainanensis* is only found in Changjiang Li Autonomous County and Dongfang City, Hainan Province based on our current knowledge and may represent a species endemic to Hainan, China.

**Ecology**. *Aleuritopteris hainanensis* is observed at the rock crevices of limestone, growing at an elevation of approximately 300–600 m a.s.l.

**Etymology**. The species is native to Hainan Province, southern China.

**Vernacular name**. 海南粉背蕨 (hai nan fen bei jue).

## 4 Discussion

Our study unequivocally demonstrates that the specimens from Hainan Island, previously misidentified as *A. squamosa*, represent a distinct species, which we describe here as *A. hainanensis* . This conclusion is robustly supported by a combination of morphological, phylogenetic, and genomic evidence.

Firstly, the morphological distinction between these two taxa is clear and consistent. The most critical diagnostic character lies in the scale morphology (Figure 4N,Q), a feature that has been overlooked in previous taxonomic studies of *Aleuritopteris*. This, coupled with their geographic distribution— *A. hainanensis* being endemic to the karst landscapes of Hainan Island, while *A. squamosa* is restricted to the dry‐hot valleys of Yunnan—strongly reinforces their status as independent evolutionary lineages.

Secondly, our phylogenetic analyses based on plastid data (Figure 2) confirm that *A. hainanensis* and *A. squamosa* are reciprocally monophyletic, with strong statistical support. The genetic distance between these two taxa also supports their status as independent species.

The comparative analysis of plastid genomes further corroborates their distinctness. While the overall structure and gene content are conserved, we identified a notable difference: the plastome of *A. squamosa* contains enlarged noncoding regions (ENRFOs) resulting from the insertion of approximately 5 kb of foreign DNA, a phenomenon absent in *A. hainanensis* (Figure 1). Similar plastome insertions have been reported in other ferns (Robison et al. 2018; Kim 2024). Although the functional implications of this insertion in *A. squamosa* remain unknown and were not the focus of this taxonomic study, its presence serves as a valuable, unambiguous molecular marker for differentiating these two sister species. Future investigations could explore whether this genomic structural variation contributes to the physiological differences between these species, particularly in relation to their adaptation to profoundly different habitats (karst vs. dry‐hot valleys).

In conclusion, through an integrative taxonomic approach, we have resolved the long‐standing misidentification of *Aleuritopteris* populations in Hainan. The discovery of *A. hainanensis* not only enriches our understanding of the biodiversity within this genus but also highlights the importance of critical reexamination of herbarium specimens using modern tools. The stark ecological divergence between the karst‐adapted *A. hainanensis* and the valley‐dwelling *A. squamosa* presents a compelling system for future research into the adaptive evolution of ferns in extreme environments.

## Author Contributions

**Bin Zhang:** data curation (lead), investigation (equal), visualization (lead), writing – original draft (equal), writing – review and editing (equal). **Ting Wang:** conceptualization (equal), formal analysis (lead), investigation (equal), methodology (lead), software (lead), writing – original draft (equal), writing – review and editing (equal). **Gui‐Liang Zhang:** investigation (equal). **Guo‐Hua Zhao:** resources (supporting). **Zi‐Yue Liu:** resources (supporting). **Fa‐Guo Wang:** methodology (supporting). **Yue‐Hong Yan:** conceptualization (equal), project administration (equal), supervision (equal), writing – review and editing (equal). **Hong‐Feng Chen:** conceptualization (lead), project administration (equal), supervision (equal), writing – review and editing (equal).

## Funding

This work was supported by the National Natural Science Foundation of China (32300180), the Science and Technology Projects in Guangzhou (E33309), Sanya Yazhou Bay Science and Technology City Development & Construction Limited Company “Special Research Project on Evaluation of Local Plant Resources and Landscape Application in Sanya” (ZYHN2021‐037).

## Ethics Statement

The authors have nothing to report.

## Conflicts of Interest

The authors declare no conflicts of interest.

## Supporting information


**Figure S1:** Normalized gap distribution across the plastome of *Aleuritopteris hainanensis* based on a 1000‐bp sliding window. Two major gap peaks occur at *trnG‐UUG*–*ndhF* and *trnN‐GUU*–*ycf2*.

**Table S1:** Detailed information on plastomes used in this study.
**Table S2:** Detailed information on *rbcL* sequences used in this study.
**Table S3:** Pairwise genetic distances among *Aleuritopteris* plastomes.
**Table S4:** Pairwise genetic distances among *rbcl* sequences in *Aleuritopteris*.

**Data S1:** 25_plastid and 37_rbcL.

## Data Availability

The genome sequences generated in this study have been deposited in the National Center for Biotechnology Information (NCBI) (https://www.ncbi.nlm.nih.gov/). Accession numbers and corresponding voucher specimen information are provided in Table 1. Voucher specimens of the new species are deposited at IBSC.
