# Supplementary material for: Aleuritopteris hainanensis (Pteridaceae), a New Species From Hainan, China
Source: Ecol Evol. 2026 Feb 20;16(2):e72770. doi: 10.1002/ece3.72770 (PMC12921721; doi:10.1002/ece3.72770)
Supplement: Supplementary file 1 — Figure S1: Normalized gap distribution across the plastome of Aleuritopteris hainanensis based on a 1000‐bp sliding window. Two major gap peaks occur at trnG‐UUG–ndhF and trnN‐GUU–ycf2. [file ECE3-16-e72770-s003.docx]

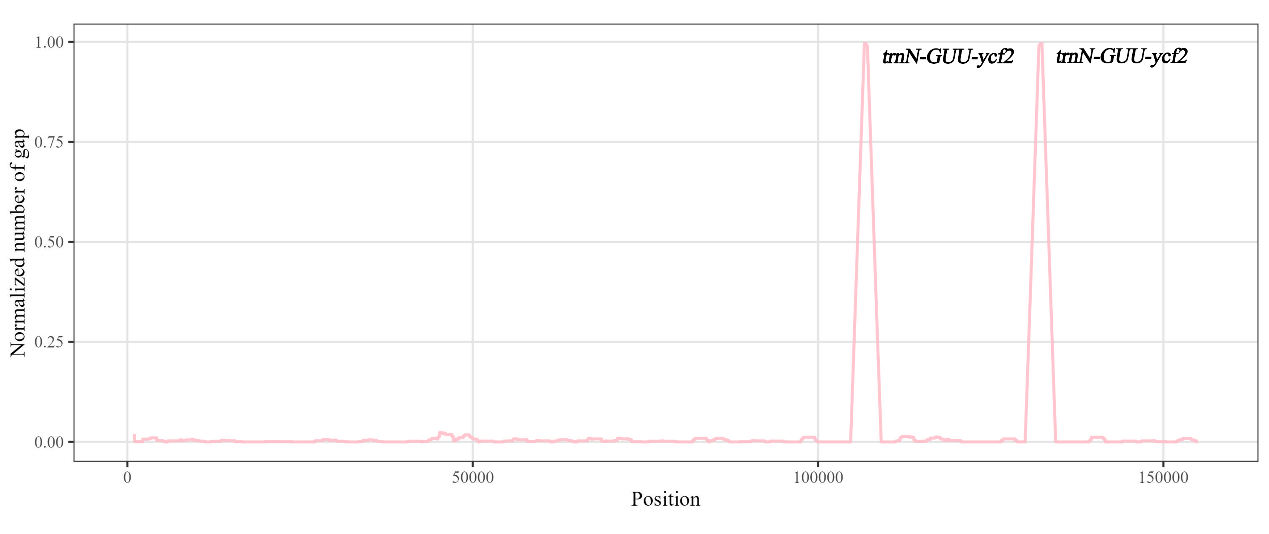


Figure S1. Normalized gap distribution across the plastome of *A. hainanensis* based on a 1000-bp sliding window. Two major gap peaks occur at *trnN-GUU*–*ycf2* .
